# Novel Genetic Variation in the KIF1A Gene Associated With Cerebellar Vermis Hypoplasia: A Case Report

**DOI:** 10.7759/cureus.94064

**Published:** 2025-10-07

**Authors:** Gabriela L Lacourt Sosa, Daniela P Torres Rodríguez, Dessire Vergés Santiago, Simon Carlo

**Affiliations:** 1 Medicine, Ponce Health Sciences University, Ponce, PRI; 2 Biochemistry/Pediatrics/Psychiatry, Ponce Health Sciences University, Ponce, PRI

**Keywords:** genetic variant, kif1a, neurodegeneration, neurodevelopment disorder, novel mutation

## Abstract

*KIF1A*-associated neuronal disorder (KAND) is a spectrum of disease caused by a genetic mutation in the *KIF1A* gene. Genetic variants in this gene have been associated with cerebellar vermis hypoplasia, as well as mild to severe symptoms, including ataxia, motor and global developmental delay, and intention tremors. Given the limited number of reported cases, ample research is necessary to effectively manage this condition.

A two-year-old girl presents with global developmental, motor, and speech delay, as well as jerky movements, possible athetosis, hand tremors, ataxia, vertical nystagmus, and muscle spasticity in lower limbs. She was referred to the clinic by her neurologist due to abnormal findings in her MRI, which showed cerebellar vermis hypoplasia. These results, together with her symptoms, suggested a genetic basis, for which a chromosomal microarray (CMA) was indicated. Results for the CMA showed two regions of homozygosity, prompting a whole-exome sequencing to be performed. This test revealed an autosomal dominant heterozygous missense mutation known as c.464G>C (p.R155P). Because there was no significant family history correlating with this disease, it appears that the mutation appeared de novo. The gene affected by this mutation is the *KIF1A* gene, which is located in 2q37.3. The *KIF1A* gene is responsible for encoding the kinesin-like protein KIF1A. This protein is responsible for microtubule motor function, as well as adenosine triphosphate (ATP) binding. Even though the mutation found in this patient was classified as a variant of uncertain significance, her severe symptoms suggest a pathological correlation. Her prognosis is reserved as there is no targeted treatment available.

Due to the low incidence of this disease and the possibility of neurodegeneration in patients, more research is encouraged to develop effective disease management.

## Introduction

*KIF1A*-associated neuronal disorder (KAND) is a genetic disease that causes different degrees of neurological impairment with possible progressive neurodegeneration and neuropathic complications. With a prevalence of 1-10/100,000, its clinical manifestations are generally known to affect multiple organ systems, but their severity depends on the type of genetic mutation in each patient. Some symptoms include, but are not limited to, cerebellar atrophy, cognitive and visual impairment, spastic paraplegia, epilepsy, and peripheral neuropathy [1].

KAND occurs from hereditary or spontaneous variations in the *KIF1A* gene, which is located on chromosome 2q37.3 and encodes kinesin-like protein KIF1A. This protein is a member of the kinesin family of adenosine triphosphate (ATP)-dependent motor proteins, which are fundamental for the proper anterograde transport of presynaptic and dense-core vesicles, membranous organelles, mRNA, and protein complexes along axonal microtubules. These components that are synthesized in the cell body must be transported to the neuronal synapse for a wide range of essential functions, such as cell survival and development. In addition to cell maintenance and conservation, *KIF1A* is also responsible for cognitive processes of the brain, such as memory and learning [2].

To understand how different mutations in this gene contribute to the physiological effects seen in mammals, it is essential to first understand the molecular structure and regulation mechanisms of kinesins. Kinesins consist of dimers that have a conserved motor domain, a stalk, and a cargo-binding domain. The motor domain attaches the axonal microtubule and hydrolyzes the ATP needed for movement, while the cargo-binding domain attaches the cargo to be transported. This interaction, together with its proposed auto-inhibitory mechanism, directs its movement toward nerve endings, using the microtubules found on axons as rails so that the cargo to be transported can be moved from the cell body to the neuron terminal [3].

The process of auto-inhibition and activation of KIF1A proteins is a tightly regulated one, with two models of activation having been proposed. The first model suggests a mechanism of auto-inhibition controlled primarily by cargo binding or molecular phosphorylation. It proposes that kinesins that lack cargo are auto-inhibited by existing in a folded state where the tail is free to interact with the motor domain, preventing ATP hydrolysis and, thus, active movement. The second model proposes a conformational change from an inactive monomeric state to an active dimeric state. This change is thought to be induced exclusively by cargo binding to the kinesin tail. In this model, the tail portion of the protein is not directly interacting with the motor domain and inhibiting ATP hydrolysis. This allows it to be readily available to bind the intended cargo and initiate the process of dimerization that allows movement along the microtubules to begin. Previous research using rat models to test the auto-inhibition mechanisms of KIF1A proteins shows that the protein already exists in a dimer form when it is auto-inhibited, supporting the model of cargo-induced activation without the need for conformational changes [4].

After understanding how kinesins are able to transport essential molecules through axonal microtubules, special attention must be paid to the different mutations that have been examined because their pathological manifestation depends on the specific type of mutation in the *KIF1A* gene. Previous studies have shown that the degree of severity in clinical symptoms depends greatly on whether the genetic mutation is a gain-of-function (GoF) or a loss-of-function (LoF) mutation, as well as the specific location of the mutation in the gene. GoF mutations have been shown to cause the hyperactivation of the protein, leading to excessive motility and synaptic vesicle accumulation at the nerve terminal. On the contrary, LoF mutations reduced the motility of the protein, leading to the accumulation of synaptic vesicles in the cell bodies, rather than at the terminals. These studies associate LoF mutations with more severe phenotypes that include hereditary spastic paraplegia and intellectual disability, while GoF mutations show milder phenotypes with hereditary spastic paraplegia but no intellectual disability [4].

We present a case of a patient with a variant of uncertain significance in the *KIF1A* gene showing symptoms of developmental delay with visual disturbances, as well as neuromuscular damage.

## Case presentation

**Figure 1:**
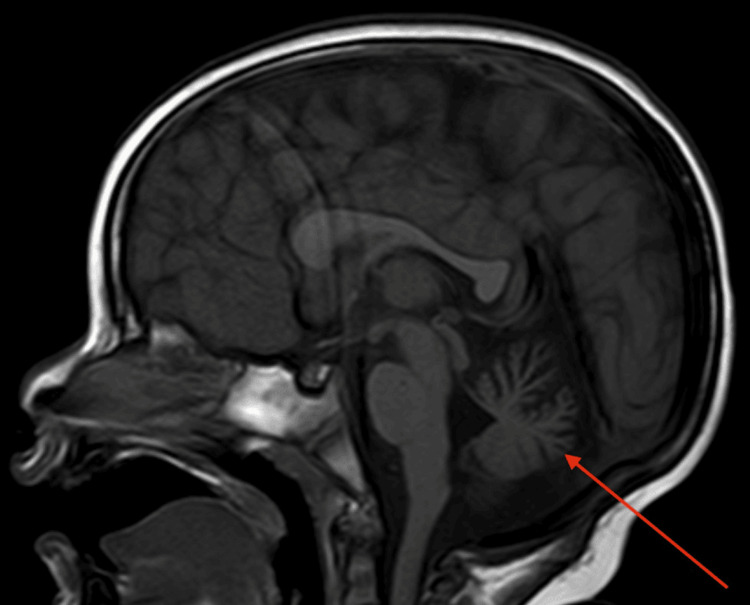
Brain MRI scan of the patient. Red arrow: the cerebellum showing vermis hypoplasia.

A two-year-old Puerto Rican girl presented with abnormal movements and developmental delay. She was evaluated by a neurologist who made an initial diagnosis of global developmental delay and gross motor delay, as well as language delay. A previous brain MRI showed cerebellar vermis hypoplasia (Figure 1).

Upon further evaluation, she showed the hypertonia of bilateral lower extremities and bilateral upgoing toes on plantar reflex, which were indicative of a positive Babinski sign (Figure 1). Additionally, she demonstrated vertical nystagmus, head oscillations, and hand tremors when initiating movement to grab objects. These symptoms directed the diagnosis toward an upper motor neuron lesion, as well as a cerebellum lesion. Subsequently, a diagnosis of cerebral palsy was suggested. The final diagnoses by her neurologist were other disorders of psychological development, specific disorder of motor function, mixed-receptive-expressive language disorder, unspecified tremor, unspecified nystagmus, unspecified abnormal involuntary movements, and transient alteration of awareness. She was referred to a geneticist for the evaluation of possible causes of the disease.

Upon evaluation, additional symptoms of jerky movements, possible athetosis, ataxia, muscle spasticity in lower limbs, and recurrent infections were noted. Due to this clinical presentation and the absence of abnormal metabolic panels or any contributing family history, a chromosomal microarray (CMA) was ordered.

Results for this test showed two regions of allelic homozygosity (ROH) with a combined length of approximately 16 Mb, including regions of chromosomes 1 and 7. Next, a whole-exome sequencing was ordered, which reported a variant of uncertain clinical significance on gene *KIF1A*, specifically c.464G>C (p.R155P), causing the arginine at amino acid position 155 to be replaced by a proline. This variation was described as autosomal dominant, heterozygous, and related to KANDs.

## Discussion

**Figure 2:**
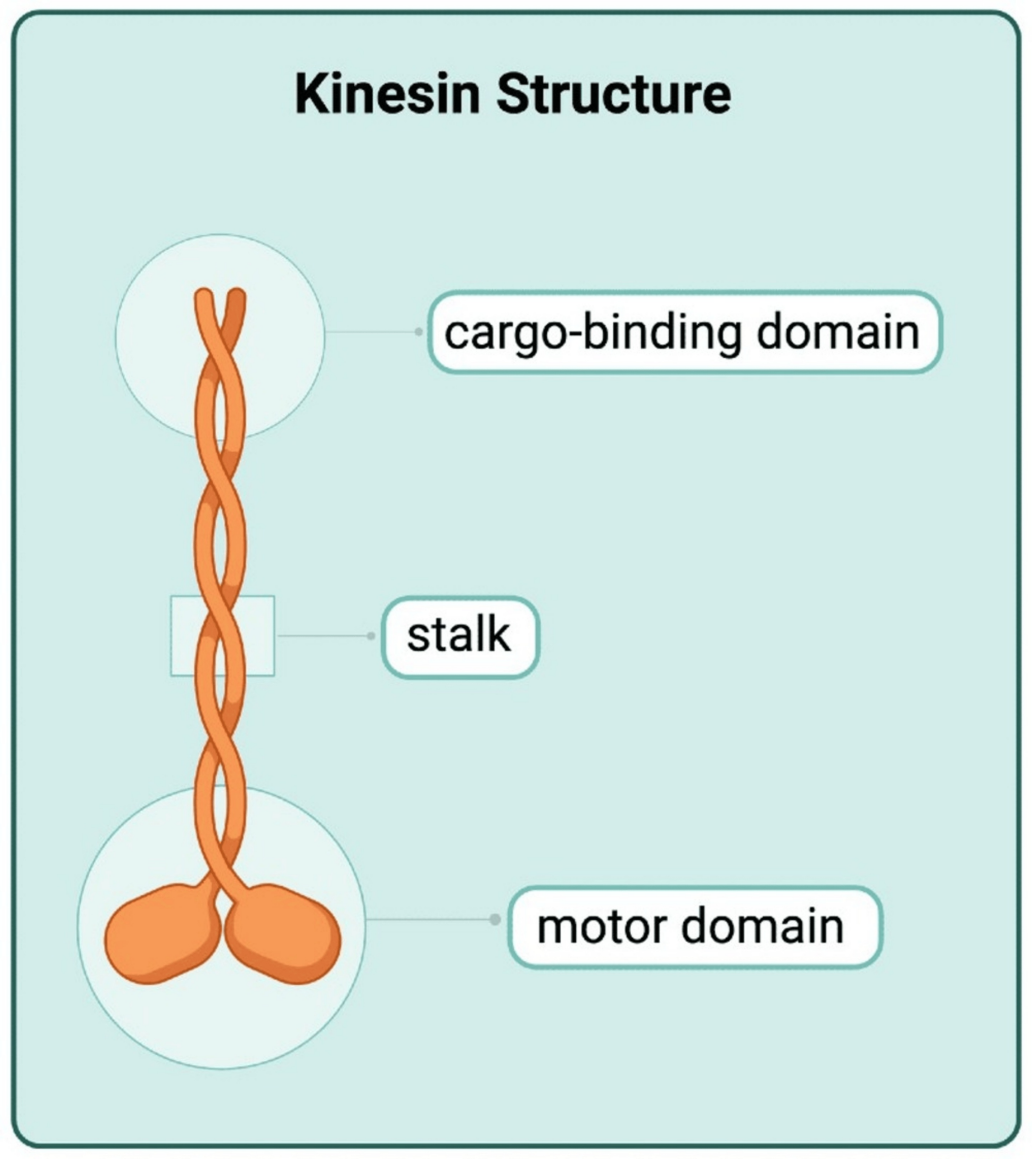
Schematic representation of the KIF1A protein structure. Created in BioRender by Lacourt Sosa GL (2025) (https://BioRender.com/kljj52z).

The severity of KAND’s pathological phenotype lies in the fact that adequate neurological function depends on this tightly regulated system of transport and communication between axons, which, in turn, has been shown to largely depend on kinesin proteins with KIF1A playing a central role (Figure 2).

The specific degree of the disease’s pathogenicity has been shown to be determined by the type of mutation, with previous case studies showing variable phenotypes that correlate with different mutations in the same gene. However, it is believed that mutations affecting the motor domain of the KIF1A protein are implicated in the highest level of clinical manifestations, whereas mutations affecting other regions of the protein have been shown to be nonpathogenic [5].

**Figure 3:**
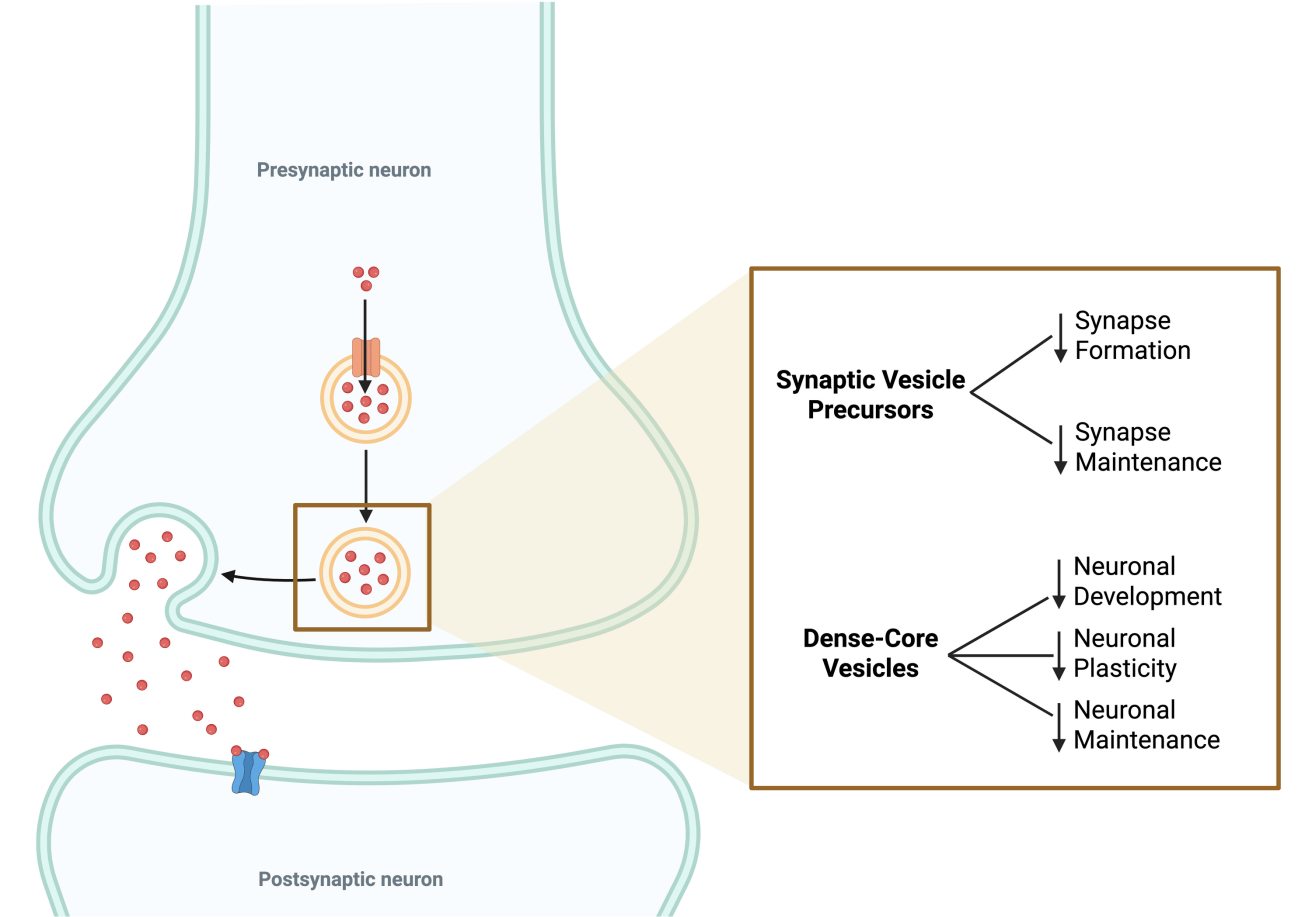
Schematic representation of the neuronal synapse and the effect of transport components on neurological status. Created in BioRender by Lacourt Sosa GL (2025) (https://BioRender.com/qzjw2on).

Previous research regarding the effect of KIF1A defects on the cellular mechanism of individuals reveals how different errors in microtubular transport lead to different cellular phenotypes. This variation in clinical manifestations occurs due to the importance and specificity of each cargo transported by the kinesin (Figure 3).

The defective transport and localization of synaptic vesicle precursors have been shown to decrease synapse formation and maintenance, while defective dense-core vesicles affect neuronal development, maintenance, and plasticity. Consequently, patients with genetic variants in the *KIF1A* gene can show varying degrees of intellectual disability, as well as neurodegeneration. Another important substance that is affected in genetic mutations of this sort is brain-derived neurotrophic factor. This neurotrophin has been known to be expressed in critical areas of the brain, such as the hippocampus, cortex, pyramidal tracts, and cerebellum, which are thought to be involved in hereditary spastic paraplegia-like disorders. In addition to brain-derived neurotrophic factor, other substances that are essential for neurological function are vesicular monoamine transporter 2 (VME2), netrin-1, and autophagy-related protein 9 (ARP9). VME2 is a protein transported via dense-core vesicles that is involved in the transport and accumulation of monoamines into synaptic vesicles, with norepinephrine, dopamine, and serotonin being the main ones affected. On the other hand, netrin-1 is a protein involved in cortical and cerebellar migration, while ARP9 is responsible for protein clearance and damaged organelle recycling. Previous research has shown that ARP9 knockout mice have an accelerated rate of neurodegeneration, confirming the chemical’s vital function [6]. This could directly correlate with the phenotype of our subject, which shows signs of muscle spasticity and cerebellar atrophy that could potentially be caused by the improper transport of these chemicals.

The whole exome sequencing report performed on our subject revealed a unique genetic variant in the *KIF1A* gene that had not been previously reported. The *KIF1A* c.464G.C genetic mutation located in amino acid 155 alters the molecular properties of the protein in a great manner, affecting properties such as hydrophobicity, polarity, and pH, as well as residue weight (Table 1).

**Table 1:** Amino acid changes due to mutation in the patient.

	Wild Type	Case Variant
Trait	Arg (R)	Pro (P)
Amino Acid Name	Arginine	Proline
Polarity/Charge	+ Charge	Nonpolar
pH	Basic	Neutral
Residue Weight	156	97
Hydrophobicity Score	-4.5	-1.6
Hydrophilicity Score	3	0
Secondary Structure Propensity	Alpha Indifferent/Beta Indifferent	Strong Alpha Breaker/Strong Beta Breaker

Genetic analysis predicts it to be deleterious by in silico analysis, as the amino acid change region happens to be in an area with high missense constraint. This means that the location affected in the gene has been highly conserved in available vertebrate species due to probable low tolerance to changes that lead to highly disrupted proteins. These defective proteins are not compatible with evolutionary pressures, as they confer a survival disadvantage to the organism bearing the genetic mutation. Deleterious mutations of this kind have been shown to be removed from the genetic pool through the process of negative selection, making them extremely rare but also severely pathogenic. Although these analyses suggest a deleterious effect of the variant, future studies including segregation analyses, functional assays, and advanced computational modeling would aid to officially classify the variant as pathogenic under American College of Medical Genetics and Genomics (ACMG)/Association for Molecular Pathology (AMP) guidelines.

In addition to the mutation in the *KIF1A* gene that is correlated to KAND, the CMA performed on our subject revealed a region of allelic homozygosity in chromosomes 1 and 7, which could be indicative of familial consanguinity [7]. The genetic analysis states that even though this result is not sufficient for a definitive diagnosis of any specific condition, it does raise the risk of a recessive Mendelian disorder with a causative genetic variant in this specific region. This finding is worth analyzing in conjunction with the variant in the *KIF1A* gene as a possible cause for the subject’s pathogenic condition, as no additional analysis was conducted to identify potentially pathogenic recessive variants within these ROHs.

## Conclusions

The rarity of the genetic mutation, as well as the distinct clinical presentation of the subject in this case report, exemplifies the need for more extensive research regarding the specific mechanism of KANDs. More information is needed to identify whether the damage caused in the kinesin-like KIF1A protein is due to defects in its ATP hydrolysis ability, protein structure, or its cargo-binding abilities. There is optimism that ascertaining the specific defect in each individual patient’s KIF1A protein product will help tailor a more assertive and effective line of treatment for KAND.
